# (*Z*)-Ethyl 3-(4-chloro­phen­yl)-2-cyano-3-(2,6-difluoro­benzamido)acrylate

**DOI:** 10.1107/S1600536808034818

**Published:** 2008-11-08

**Authors:** Zhang Dehua, Zhang Xiaoyan

**Affiliations:** a Department of Chemistry and Environmental Engineering, Hubei Normal University, Huangshi 435002, People’s Republic of China; b School of Mathematics and Physics, Huangshi Institute of Tecnology, Huangshi 435003, People’s Republic of China

## Abstract

The title compound, C_19_H_13_ClF_2_N_2_O_3_, was prepared by the reaction of (*Z*)-ethyl 3-amino-3-(4-chloro­phen­yl)-2-cyano­acrylate and 2,6-difluoro­benzoyl chloride. The dihedral angle between the chloro­benzene and fluoro­benzene rings is 37.0 (1)°. The ethyl group is disordered over two positions [occupancies = 0.52 (2):0.48 (2)]. In addition to intra­molecular N—H⋯O and N—H⋯F hydrogen bonds, the crystal packing shows the mol­ecules to be connected by inter­molecular C—H⋯O and C—H⋯N hydrogen bonds.

## Related literature

The title compound is useful as an inhibitor of *Pyricularia oryzae*, *Rhizoctonia solani*, *Botrytis cinerea* and *Gibberella zeae*, see: Heller *et al.* (2004 ▶); Creagh & Hubbell (1992 ▶); Ibers & Hamilton (1964 ▶).
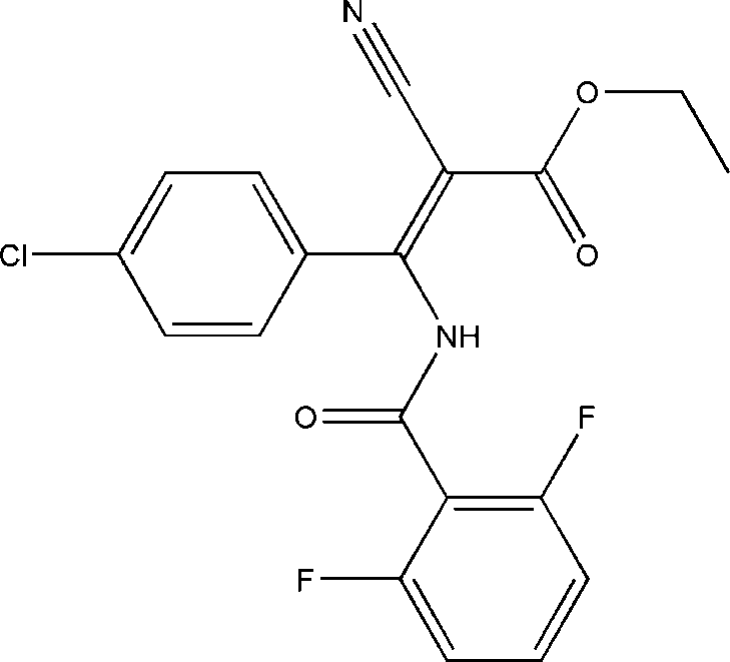


## Experimental

### 

#### Crystal data

C_19_H_13_ClF_2_N_2_O_3_
*M* *_r_* = 390.76
Triclinic, 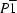

*a* = 8.919 (5) Å
*b* = 9.7560 (6) Å
*c* = 11.2717 (7) Å
α = 91.9710 (10)°
β = 110.0940 (10)°
γ = 91.4490 (10)°
*V* = 919.9 (5) Å^3^
*Z* = 2
Mo *K*α radiation
μ = 0.25 mm^−1^
*T* = 298 (2) K
0.23 × 0.20 × 0.10 mm

#### Data collection

Bruker SMART CCD area-detector diffractometer
Absorption correction: none
7196 measured reflections
3556 independent reflections
2524 reflections with *I* > 2σ(*I*)
*R*_int_ = 0.053

#### Refinement

*R*[*F* ^2^ > 2σ(*F* ^2^)] = 0.052
*wR*(*F* ^2^) = 0.154
*S* = 1.10
3556 reflections
265 parameters
6 restraints
H-atom parameters constrained
Δρ_max_ = 0.22 e Å^−3^
Δρ_min_ = −0.31 e Å^−3^

### 

Data collection: *SMART* (Bruker, 1998 ▶); cell refinement: *SAINT* (Bruker, 1999 ▶); data reduction: *SAINT*; program(s) used to solve structure: *SHELXS97* (Sheldrick, 2008 ▶); program(s) used to refine structure: *SHELXL97* (Sheldrick, 2008 ▶); molecular graphics: *SHELXTL* (Sheldrick, 2008 ▶); software used to prepare material for publication: *SHELXTL*.

## Supplementary Material

Crystal structure: contains datablocks I, global. DOI: 10.1107/S1600536808034818/fl2223sup1.cif

Structure factors: contains datablocks I. DOI: 10.1107/S1600536808034818/fl2223Isup2.hkl

Additional supplementary materials:  crystallographic information; 3D view; checkCIF report

## Figures and Tables

**Table 1:** Hydrogen-bond geometry (Å, °)

*D*—H⋯*A*	*D*—H	H⋯*A*	*D*⋯*A*	*D*—H⋯*A*
N1—H1⋯O2	0.86	2.05	2.674 (2)	129
N1—H1⋯F1	0.86	2.36	2.827 (2)	115
C18—H18*B*⋯O1^i^	0.97	2.58	2.990 (7)	106
C10—H10⋯N2^ii^	0.93	2.62	3.302 (3)	131
C5—H5⋯N2^iii^	0.93	2.59	3.432 (3)	150

Symmetry codes: (i) 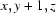; (ii) 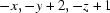; (iii) 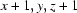.

**Fig. 1.**
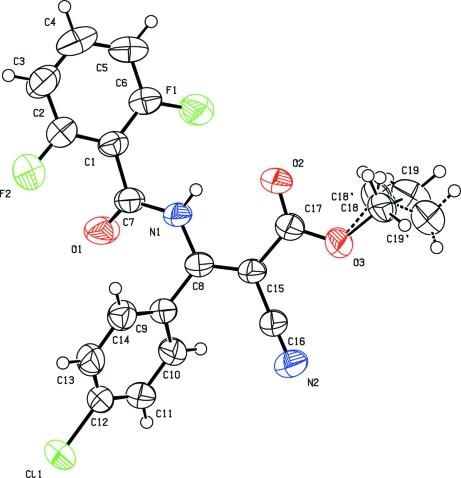
The molecular structure of the title compound, showing 30% probability displacement ellipsoids and the atom-numbering scheme.

**Fig. 2.**
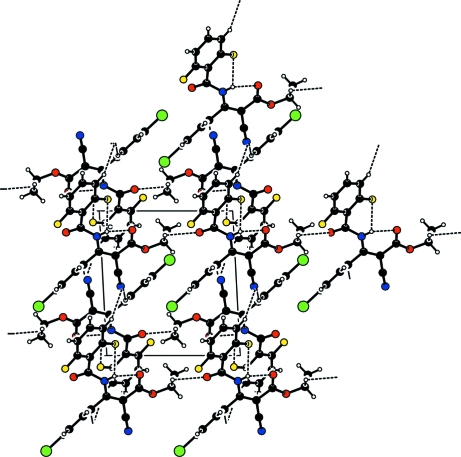
The packing of the title compound, viewed down the c axis.

## supplementary crystallographic information

### Comment

Recently, 2-cyanoacrylates have been in widespread used as agrochemicals because of their unique mechanism of action and good environmental profiles. The title compound is useful as an inhibitor of Pyricularia oryzae, Rhizoctonia solani, Botrytis cinerea and Gibberella zeae (Heller *et al.*, 2004; Creagh & Hubbell, 1992; Ibers & Hamilton, 1964).

In the title compound(Fig.1),all bond lengths and angles are unexceptional.The planar chlorobenzene ring is approximately perpendicular to the fluorobenzene ring with a dihedral angle of 37.0 (1)°. The ethyl group is disordered over two positions occupancies (0.52 (2):0.48 (2)).The molecular conformation is stabilized by C—H···O and N—H···O hydrogen bonds (Table 1). The crystal packing is governed by additional N—H···O and N—H···F Interactions (Fig. 2).

### Experimental

To a solution of (*Z*)-ethyl 3-amino-3-(4-chlorophenyl)-2-cyanoacrylate (1.25 g,0.0050 mol) in CH_2_Cl_2_(18 ml), 2,6-difluorobenzoyl chloride (2.65 g,0.015 mol) was added. Subsequently, Et_3_N(1.52 g,0.015 mol) waa dropped into the solution under stirring. Then, the reaction mixture was heated to reflux and stirred for 4 h and then cooled to room temperature. The reaction solution was filtered off and some white solid was separated. The organic phase was washed with water and then dried over Na_2_SO_4_. After removal of the solvent, a brown dope was obtained. After column chromatography using ethylacetate/light petroleum (1:6) as the eluent. Small single crystals were grown from a solution of ethyl acetate/petroleum ether(3:1) after 45 days,at room temperature.

### Refinement

Methyl H atoms were placed in calculated positions with C—H=0.96 Å and the torsion angle was refined to fit the electron density, with *UU*_iso_(H)=1.5*UU*_eq_(C). Other H atoms were placed in calculated positions with C—H =0.96 Å(methylene) and 0.93 Å(aromatic C—H), and refined in riding mode, with *U*_iso_(H)=1.2*U*_eq_(C). In the absence of significant anomalous scattering, Friedel pairs were merged.

### Crystal data

**Table:**

C_19_H_13_ClF_2_N_2_O_3_	*Z* = 2
*M**_r_* = 390.76	*F*_000_ = 400
Triclinic, *P*1	*D*_x_ = 1.411 Mg m^−^^3^
*a* = 8.919 (5) Å	Mo *K*α radiation λ = 0.71073 Å
*b* = 9.7560 (6) Å	Cell parameters from 2672 reflections
*c* = 11.2717 (7) Å	θ = 2.4–26.8º
α = 91.9710 (10)º	µ = 0.25 mm^−^^1^
β = 110.0940 (10)º	*T* = 298 (2) K
γ = 91.4490 (10)º	Block, colorless
*V* = 919.9 (5) Å^3^	0.23 × 0.20 × 0.10 mm

### Data collection

**Table:**

Bruker SMART CCD area-detector diffractometer	2524 reflections with *I* > 2σ(*I*)
Radiation source: fine-focus sealed tube	*R*_int_ = 0.053
Monochromator: graphite	θ_max_ = 26.0º
*T* = 298(2) K	θ_min_ = 1.9º
φ and ω scans	*h* = −10→9
Absorption correction: none	*k* = −12→12
7196 measured reflections	*l* = −10→13
3556 independent reflections	

### Refinement

**Table:**

Refinement on *F*^2^	Secondary atom site location: difference Fourier map
Least-squares matrix: full	Hydrogen site location: inferred from neighbouring sites
*R*[*F*^2^ > 2σ(*F*^2^)] = 0.052	H-atom parameters constrained
*wR*(*F*^2^) = 0.154	*w* = 1/[σ^2^(*F*_o_^2^) + (0.0794*P*)^2^ + 0.0184*P*] where *P* = (*F*_o_^2^ + 2*F*_c_^2^)/3
*S* = 1.10	(Δ/σ)_max_ < 0.001
3556 reflections	Δρ_max_ = 0.22 e Å^−^^3^
265 parameters	Δρ_min_ = −0.31 e Å^−^^3^
6 restraints	Extinction correction: none
Primary atom site location: structure-invariant direct methods	

### Special details

**Table:**

Geometry. All e.s.d.'s (except the e.s.d. in the dihedral angle between two l.s. planes) are estimated using the full covariance matrix. The cell e.s.d.'s are taken into account individually in the estimation of e.s.d.'s in distances, angles and torsion angles; correlations between e.s.d.'s in cell parameters are only used when they are defined by crystal symmetry. An approximate (isotropic) treatment of cell e.s.d.'s is used for estimating e.s.d.'s involving l.s. planes.
Refinement. Refinement of *F*^2^ against ALL reflections. The weighted *R*-factor *wR* and goodness of fit *S* are based on *F*^2^, conventional *R*-factors *R* are based on *F*, with *F* set to zero for negative *F*^2^. The threshold expression of *F*^2^ > σ(*F*^2^) is used only for calculating *R*-factors(gt) *etc*. and is not relevant to the choice of reflections for refinement. *R*-factors based on *F*^2^ are statistically about twice as large as those based on *F*, and *R*- factors based on ALL data will be even larger.

### Fractional atomic coordinates and isotropic or equivalent isotropic displacement parameters (Å^2^)

**Table:**

	*x*	*y*	*z*	*U*_iso_*/*U*_eq_	Occ. (<1)
C1	0.7937 (2)	0.8595 (2)	0.98955 (19)	0.0535 (5)	
C2	0.9102 (3)	0.7653 (3)	1.0005 (2)	0.0732 (7)	
C3	1.0531 (3)	0.7682 (3)	1.0992 (3)	0.0838 (8)	
H3	1.1291	0.7036	1.1024	0.101*	
C4	1.0803 (3)	0.8678 (3)	1.1917 (3)	0.0803 (8)	
H4	1.1756	0.8699	1.2601	0.096*	
C5	0.9711 (3)	0.9653 (3)	1.1869 (2)	0.0751 (7)	
H5	0.9911	1.0336	1.2506	0.090*	
C6	0.8309 (3)	0.9595 (2)	1.0850 (2)	0.0595 (6)	
C7	0.6359 (3)	0.8440 (2)	0.8831 (2)	0.0559 (5)	
C8	0.4458 (2)	0.97457 (19)	0.71787 (19)	0.0488 (5)	
C9	0.3884 (2)	0.85359 (19)	0.62766 (19)	0.0502 (5)	
C10	0.2296 (3)	0.8088 (2)	0.5863 (2)	0.0622 (6)	
H10	0.1586	0.8521	0.6180	0.075*	
C11	0.1759 (3)	0.7012 (2)	0.4989 (2)	0.0750 (8)	
H11	0.0695	0.6703	0.4725	0.090*	
C12	0.2800 (4)	0.6397 (2)	0.4511 (2)	0.0769 (8)	
C13	0.4383 (3)	0.6827 (2)	0.4905 (2)	0.0755 (7)	
H13	0.5082	0.6400	0.4573	0.091*	
C14	0.4923 (3)	0.7894 (2)	0.5793 (2)	0.0621 (6)	
H14	0.5994	0.8184	0.6069	0.074*	
C15	0.3749 (2)	1.09761 (19)	0.6912 (2)	0.0513 (5)	
C16	0.2483 (2)	1.1126 (2)	0.5730 (2)	0.0560 (5)	
C17	0.4278 (3)	1.2223 (2)	0.7746 (2)	0.0620 (6)	
C18	0.3811 (12)	1.4663 (7)	0.7837 (10)	0.068 (2)	0.523 (18)
H18A	0.3563	1.5287	0.7150	0.081*	0.523 (18)
H18B	0.4946	1.4746	0.8316	0.081*	0.523 (18)
C19	0.2853 (15)	1.4949 (11)	0.8666 (11)	0.092 (3)	0.523 (18)
H19A	0.1736	1.4875	0.8169	0.138*	0.523 (18)
H19B	0.3120	1.5860	0.9044	0.138*	0.523 (18)
H19C	0.3087	1.4296	0.9317	0.138*	0.523 (18)
H18C	0.3968	1.4029	0.9188	0.105*	0.477 (18)
H18D	0.4638	1.4940	0.8343	0.105*	0.477 (18)
H19D	0.1927	1.5423	0.7192	0.136*	0.477 (18)
H19E	0.2613	1.6103	0.8561	0.136*	0.477 (18)
H19F	0.1498	1.4776	0.8294	0.136*	0.477 (18)
Cl1	0.21456 (12)	0.50444 (8)	0.33944 (8)	0.1331 (5)	
F1	0.72205 (17)	1.05606 (16)	1.07956 (13)	0.0850 (5)	
F2	0.8855 (2)	0.66854 (19)	0.90845 (19)	0.1238 (7)	
N1	0.57876 (19)	0.96329 (16)	0.82386 (16)	0.0544 (5)	
H1	0.6319	1.0382	0.8567	0.065*	
N2	0.1517 (2)	1.1285 (2)	0.4780 (2)	0.0741 (6)	
O1	0.5673 (2)	0.73410 (15)	0.85157 (17)	0.0825 (6)	
O2	0.5482 (2)	1.23045 (16)	0.86745 (17)	0.0837 (6)	
O3	0.3324 (2)	1.32363 (15)	0.73535 (19)	0.0887 (6)	

### Atomic displacement parameters (Å^2^)

**Table:**

	*U*^11^	*U*^22^	*U*^33^	*U*^12^	*U*^13^	*U*^23^
C1	0.0522 (12)	0.0567 (11)	0.0446 (11)	−0.0027 (9)	0.0076 (9)	0.0076 (9)
C2	0.0721 (16)	0.0698 (14)	0.0661 (16)	0.0128 (12)	0.0083 (13)	0.0047 (12)
C3	0.0652 (16)	0.0906 (18)	0.082 (2)	0.0167 (13)	0.0062 (15)	0.0179 (16)
C4	0.0527 (14)	0.111 (2)	0.0633 (16)	−0.0062 (14)	0.0010 (12)	0.0252 (15)
C5	0.0707 (16)	0.0961 (18)	0.0465 (14)	−0.0138 (14)	0.0067 (12)	−0.0015 (12)
C6	0.0531 (13)	0.0744 (14)	0.0479 (12)	−0.0006 (10)	0.0138 (10)	0.0029 (10)
C7	0.0562 (12)	0.0551 (11)	0.0467 (12)	−0.0040 (9)	0.0056 (10)	0.0024 (9)
C8	0.0412 (10)	0.0534 (10)	0.0477 (12)	−0.0037 (8)	0.0106 (9)	−0.0004 (9)
C9	0.0500 (11)	0.0491 (10)	0.0445 (11)	0.0017 (8)	0.0076 (9)	0.0003 (8)
C10	0.0521 (12)	0.0533 (11)	0.0711 (15)	0.0009 (9)	0.0098 (11)	−0.0098 (10)
C11	0.0645 (15)	0.0569 (13)	0.0773 (17)	0.0016 (11)	−0.0080 (13)	−0.0112 (12)
C12	0.0947 (19)	0.0522 (12)	0.0568 (15)	0.0208 (12)	−0.0091 (13)	−0.0072 (11)
C13	0.095 (2)	0.0717 (15)	0.0563 (15)	0.0297 (14)	0.0200 (14)	−0.0033 (12)
C14	0.0616 (14)	0.0673 (13)	0.0572 (14)	0.0130 (11)	0.0197 (11)	0.0023 (11)
C15	0.0438 (11)	0.0514 (11)	0.0498 (12)	−0.0033 (8)	0.0056 (9)	−0.0014 (9)
C16	0.0464 (12)	0.0516 (11)	0.0612 (14)	0.0009 (9)	0.0076 (11)	−0.0018 (10)
C17	0.0554 (13)	0.0512 (11)	0.0671 (15)	−0.0036 (10)	0.0063 (12)	−0.0033 (10)
C18	0.084 (4)	0.037 (3)	0.067 (5)	−0.004 (3)	0.007 (4)	0.001 (3)
C19	0.102 (8)	0.082 (6)	0.082 (6)	−0.018 (5)	0.022 (5)	−0.025 (5)
Cl1	0.1606 (9)	0.0804 (5)	0.0965 (6)	0.0416 (5)	−0.0328 (6)	−0.0442 (4)
F1	0.0830 (10)	0.0996 (10)	0.0646 (9)	0.0167 (8)	0.0164 (8)	−0.0169 (8)
F2	0.1283 (15)	0.1036 (12)	0.1083 (14)	0.0450 (11)	0.0014 (11)	−0.0327 (11)
N1	0.0465 (10)	0.0488 (9)	0.0534 (11)	−0.0040 (7)	−0.0005 (8)	−0.0004 (8)
N2	0.0581 (12)	0.0760 (13)	0.0698 (14)	0.0017 (10)	−0.0014 (11)	0.0026 (10)
O1	0.0875 (12)	0.0556 (9)	0.0744 (12)	−0.0176 (8)	−0.0097 (9)	0.0096 (8)
O2	0.0777 (11)	0.0612 (9)	0.0783 (12)	−0.0010 (8)	−0.0145 (10)	−0.0155 (8)
O3	0.0693 (11)	0.0497 (9)	0.1138 (15)	0.0055 (7)	−0.0093 (10)	−0.0193 (9)

### Geometric parameters (Å, °)

**Table:**

C1—C6	1.372 (3)	C11—C12	1.365 (4)
C1—C2	1.382 (3)	C11—H11	0.9300
C1—C7	1.504 (3)	C12—C13	1.376 (4)
C2—F2	1.335 (3)	C12—Cl1	1.734 (2)
C2—C3	1.373 (3)	C13—C14	1.374 (3)
C3—C4	1.355 (4)	C13—H13	0.9300
C3—H3	0.9300	C14—H14	0.9300
C4—C5	1.368 (4)	C15—C16	1.435 (3)
C4—H4	0.9300	C15—C17	1.474 (3)
C5—C6	1.375 (3)	C16—N2	1.139 (3)
C5—H5	0.9300	C17—O2	1.214 (3)
C6—F1	1.358 (3)	C17—O3	1.308 (3)
C7—O1	1.200 (2)	C18—O3	1.475 (6)
C7—N1	1.382 (3)	C18—C19	1.491 (8)
C8—C15	1.365 (3)	C18—H18A	0.9700
C8—N1	1.374 (2)	C18—H18B	0.9700
C8—C9	1.489 (3)	C19—H19A	0.9600
C9—C14	1.379 (3)	C19—H19B	0.9600
C9—C10	1.384 (3)	C19—H19C	0.9600
C10—C11	1.372 (3)	N1—H1	0.8600
C10—H10	0.9300		
			
C6—C1—C2	115.1 (2)	C12—C11—H11	120.3
C6—C1—C7	124.45 (19)	C10—C11—H11	120.3
C2—C1—C7	120.34 (19)	C11—C12—C13	121.0 (2)
F2—C2—C3	117.8 (2)	C11—C12—Cl1	120.3 (2)
F2—C2—C1	118.6 (2)	C13—C12—Cl1	118.7 (2)
C3—C2—C1	123.6 (2)	C14—C13—C12	119.5 (2)
C4—C3—C2	118.2 (3)	C14—C13—H13	120.3
C4—C3—H3	120.9	C12—C13—H13	120.3
C2—C3—H3	120.9	C13—C14—C9	120.3 (2)
C3—C4—C5	121.6 (2)	C13—C14—H14	119.9
C3—C4—H4	119.2	C9—C14—H14	119.9
C5—C4—H4	119.2	C8—C15—C16	119.88 (17)
C4—C5—C6	118.1 (2)	C8—C15—C17	123.52 (18)
C4—C5—H5	121.0	C16—C15—C17	116.48 (17)
C6—C5—H5	121.0	N2—C16—C15	177.2 (2)
F1—C6—C1	118.17 (18)	O2—C17—O3	124.01 (19)
F1—C6—C5	118.3 (2)	O2—C17—C15	123.6 (2)
C1—C6—C5	123.5 (2)	O3—C17—C15	112.34 (18)
O1—C7—N1	123.20 (19)	O3—C18—C19	103.8 (6)
O1—C7—C1	121.33 (19)	O3—C18—H18A	111.0
N1—C7—C1	115.46 (16)	C19—C18—H18A	111.0
C15—C8—N1	120.33 (16)	O3—C18—H18B	111.0
C15—C8—C9	120.59 (17)	C19—C18—H18B	111.0
N1—C8—C9	118.91 (17)	H18A—C18—H18B	109.0
C14—C9—C10	119.25 (19)	C8—N1—C7	126.72 (16)
C14—C9—C8	119.85 (19)	C8—N1—H1	116.6
C10—C9—C8	120.82 (18)	C7—N1—H1	116.6
C11—C10—C9	120.6 (2)	C17—O3—C18	121.6 (5)
C11—C10—H10	119.7	C17—O3—C18'	110.6 (4)
C9—C10—H10	119.7	C18—O3—C18'	26.1 (5)
C12—C11—C10	119.4 (2)		

### Hydrogen-bond geometry (Å, °)

**Table:**

*D*—H···*A*	*D*—H	H···*A*	*D*···*A*	*D*—H···*A*
N1—H1···O2	0.86	2.05	2.674 (2)	129
N1—H1···F1	0.86	2.36	2.827 (2)	115
C18—H18B···O1^i^	0.97	2.58	2.990 (7)	106
C10—H10···N2^ii^	0.93	2.62	3.302 (3)	131
C5—H5···N2^iii^	0.93	2.59	3.432 (3)	150

Symmetry codes: (i) *x*, *y*+1, *z*; (ii) −*x*, −*y*+2, −*z*+1; (iii) *x*+1, *y*, *z*+1.
